# Relative efficacy of systemic treatments for patients with relapsed/refractory chronic lymphocytic leukemia: a network meta-analysis according to 17p deletion/*TP53* mutations

**DOI:** 10.1007/s44313-024-00038-2

**Published:** 2025-01-02

**Authors:** Jinchul Kim, Jinhyun Cho, Joo Han Lim, Moon Hee Lee

**Affiliations:** Department of Hematology-Oncology, Inha University College of Medicine and Hospital, 7-206 Third Street, Shinheung-Dong Jung-Gu, Incheon, Republic of Korea

**Keywords:** Relapsed/refractory CLL, Network meta-analysis, Venetoclax, Zanubrutinib

## Abstract

**Purpose:**

This network meta-analysis aimed to evaluate the relative efficacy of systemic treatments in patients with relapsed/refractory chronic lymphocytic leukemia (R/R CLL), focusing on key genetic mutations, specifically the 17p deletion and *TP53* mutations.

**Methods:**

We conducted a systematic literature review to identify all publicly available randomized controlled trials (RCTs) using PubMed, EMBASE, the Cochrane database, and meeting abstracts published through December 2023. A Bayesian network meta-analysis was performed to estimate the hazard ratios (HRs) for progression-free survival (PFS) with 95% confidence intervals (CIs) and to determine the ranking of the included regimens.

**Results:**

Twelve trials involving 4,437 patients and 13 treatment options were included in the meta-analysis. Venetoclax plus rituximab and zanubrutinib emerged as the most effective treatments for the overall R/R CLL population, showing the lowest PFS HR (HR 0.62, 95% CI 0.32–1.20 and HR 0.65, 95% CI 0.49–0.86, respectively) versus ibrutinib, and were ranked as the best agent (surface under the cumulative ranking curve [SUCRA] value of both 90%, respectively) among the included drugs. In the 17p deletion/*TP53* mutation subgroup, zanubrutinib demonstrated the most favorable efficacy (HR 0.52, 95% CI 0.31–0.88 versus ibrutinib) with the highest SUCRA value (97%). In patients without these mutations, venetoclax plus rituximab was the most effective (HR 0.49, 95% CI 0.26–0.94 versus ibrutinib) with a SUCRA value of 94%.

**Conclusion:**

Our findings highlight the superior efficacy of venetoclax plus rituximab and zanubrutinib for treating R/R CLL and confirm that the role of each regimen may vary depending on the clinically significant mutations.

**Supplementary Information:**

The online version contains supplementary material available at 10.1007/s44313-024-00038-2.

## Introduction

Chronic lymphocytic leukemia (CLL) is the most prevalent type of leukemia among adults in Western countries. It represents one-quarter of all leukemia cases and constitutes 1.3% of all cancer diagnoses [1]. Despite recent advances in treatment, many patients relapse or become refractory to initial therapies. Thus, there is a need to evaluate the various treatment options for relapsed/refractory (R/R) CLL [2].

The treatment landscape for R/R CLL has significantly advanced with the advent of novel targeted therapies. These include Bruton tyrosine kinase (BTK) inhibitors, such as ibrutinib, acalabrutinib and zanubrutinib, phosphatidylinositol 3-kinase (PI3K) inhibitors, such as idelalisib and duvelisib, as well as the B-cell lymphoma 2 (BCL-2) inhibitor venetoclax [3]. Although these treatments have demonstrated improved outcomes in R/R CLL patients, there is no established standard of care [4]. The selection of an appropriate regimen depends on various factors, including prior treatment history, response to previous therapies, potential drug toxicities, presence of comorbidities, and important genomic alterations, such as 17p deletion or *TP53* mutation.

Given the diversity of available treatments and the lack of head-to-head comparisons of all options, there is a critical need for a comprehensive evaluation of their relative efficacy. Network meta-analysis provides a robust methodological framework for simultaneously comparing multiple interventions, even in the absence of direct comparative trials [5]. By synthesizing data from multiple randomized controlled trials, we sought to provide a robust comparison of available therapies, including BTKis, venetoclax-based regimens, and other novel agents. In this study, a comprehensive network meta-analysis was conducted to compare the efficacies of various r/r CLL treatments. To reduce heterogeneity and provide clinically relevant information, we first analyzed the entire population, followed by separate analyses for groups with and without 17p deletions or *TP53* mutations.

## Materials and methods

### Systematic literature review

We conducted a comprehensive literature search from inception to December 1, 2023. We searched PubMed, EMBASE, and the Cochrane Central Register of Controlled Trials using the keywords “chronic lymphocytic leukemia” and “randomized controlled trial” (see Supplementary Methods for detailed search strategies). The search was restricted to human trials, with no language limitations. Additionally, we scanned meeting abstracts from the American Society of Clinical Oncology, American Society of Hematology, European Society for Medical Oncology, and European Hematology Association, as well as references of relevant articles, to identify further pertinent studies.

### Eligibility criteria

Randomized controlled trials evaluating the efficacy of systemic treatment in patients with R/R CLL were selected for the network meta-analysis. Additional inclusion criteria included full-text articles and primary outcome reports. Studies with the following characteristics were excluded: (1) published before 2000, (2) those studying the efficacy of hematopoietic stem cell transplantation only, (3) the efficacy of maintenance treatment, or (4) the efficacy of drugs after initial treatment, (5) studies that were not properly terminated for various reasons, and (6) those that did not form a network framework with trials that studied ibrutinib, since ibrutinib is considered the cornerstone treatment for CLL. Two independent reviewers (J.K. and J.C.) selected and reviewed all the screened articles.

### Data extraction

Two authors independently extracted the following information from each selected study (J.K. and M.H.L.): trial name (if available)/identifier, name of the first author, published journal, publication year, trial phase, country in which the trial was conducted, intervention and control treatments, and median follow-up duration. Clinical information on median age (range), median number of previous therapies (range), total number of patients, proportion of male patients, Eastern Cooperative Oncology Group (ECOG) performance score 2, bulky disease, Rai stage III/IV, del(17p), del(11q), *TP53* mutation, immunoglobulin heavy-chain variable region gene (*IGHV*) mutation, and complex karyotype were also collected. The other two authors (J.C. and J.H.L.) resolved any discrepancies in the extracted data.

### Data synthesis and analysis

The entire study population was initially analyzed. To minimize heterogeneity and deliver clinically relevant insights, we conducted separate analyses based on the presence or absence of 17p deletion or *TP53* mutation, as treatment strategies for CLL differ significantly based on these genetic abnormalities.

In our study, a network meta-analysis using a fixed-effects model was considered appropriate because the included studies were well-designed randomized trials that shared key similarities, such as patient characteristics with significant genomic features, lines of treatment, and outcome measurements.

Network meta-analysis was performed using hazard ratios (HRs) and corresponding 95% confidence intervals (CIs) for progression-free survival (PFS), the primary outcome, within a Bayesian framework. Non-informative priors were established for relative treatment effects. Four Markov chains were automatically generated, each with 5,000 adaptations and 20,000 iterations. The outcomes, which were the relative effects of the included treatments, reported as HRs with 95% credible intervals (CIs) for PFS, were calculated. To estimate the overall rankings of all included treatments, the surface under the cumulative ranking curve (SUCRA) was calculated for each treatment. SUCRA represents the summation of each treatment’s relative ranking probabilities, indicating the likelihood of each intervention achieving a possible rank concerning its relative efficacy. SUCRA values range from 0 (worst) to 1 (best), with the treatment with the highest SUCRA value considered the best [6]. The JAGS and the GeMTC package [7] were used in R studio software (version 4.0.3, R Foundation).

## Results

### Literature search

**Fig. 1:**
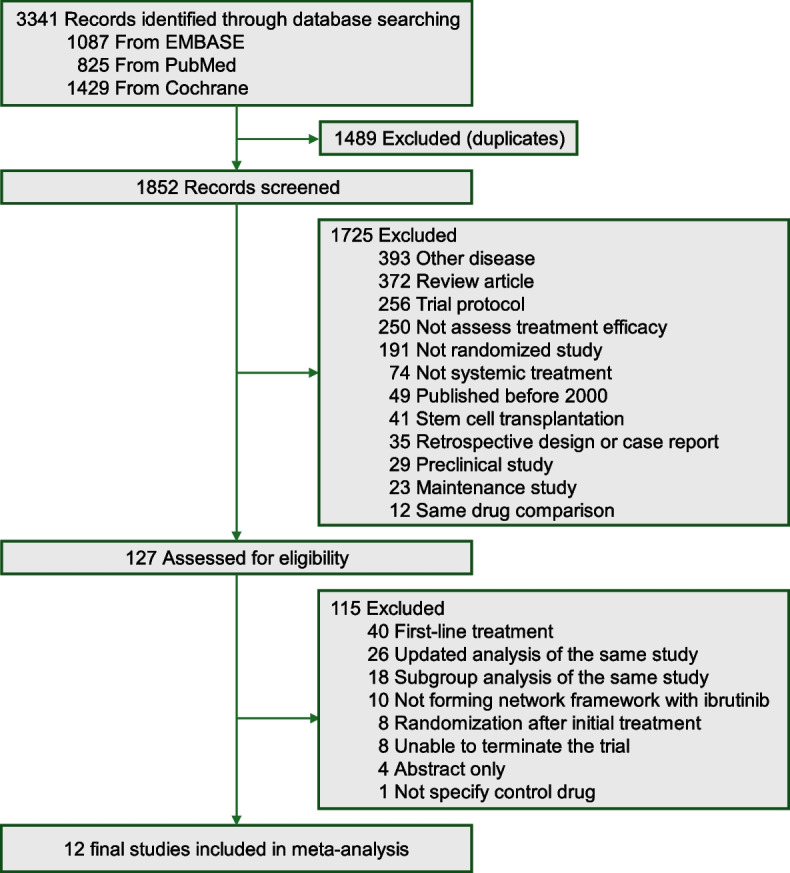
Trial selection flow

A total of 3,341 records were identified through the database search. After removing 1,489 duplicates, the titles and abstracts of the remaining 1,852 records were screened. Following eligibility screening, 1,725 studies were excluded, and the full texts of 127 potentially relevant studies were reviewed. Ultimately, 12 trials [8–19] comprising 4,437 patients that met the inclusion criteria were included in the quantitative analysis (Fig. 1).

### Characteristics of included studies

The baseline characteristics of the included trials are summarized in Table 1 and Supplementary Table S1. The median age of the patients across the trials varied between 63 and 71 years, with minimal differences. The prevalence of Rai stage III/IV disease was documented in all the studies, with values ranging from 35.1% to 72.5%. The prevalence of key genetic alterations, including del(17p), del(11q), *TP53* mutations, and *IGHV* mutation status, varied widely. The median number of previous therapies was mostly one or two, indicating that most trials were conducted in second- or third-line settings, respectively. The exceptions were two studies (116 and 119) that evaluated the efficacy of idelalisib as a later-line treatment. Various treatment regimens for R/R CLL were included, primarily focusing on novel agents, such as BTK inhibitors (ibrutinib, acalabrutinib, zanubrutinib), PI3K inhibitors (idelalisib, duvelisib), and BCL-2 inhibitors (venetoclax). The control arms in most trials often included standard treatments, such as ibrutinib, rituximab, or ofatumumab. The follow-up duration ranged from 9.4 months to 40.9 months.

**Table 1:** Baseline characteristics of included studies

**Trial name**	**Authors (Trial identifier)**	**Research**	**Control**	**No. of Patients**	**Median age (range, yr)**	**Rai stage III/IV (%)**	**del(17p) (%)**	***TP53* mutated (%)**	***IGHV* unmutated (%)**	**Median No. of previous therapy (range)**
ELEVATE-RR	Byrd et al. (NCT02477696)	Acalabrutinib	Ibrutinib	533	66 (28–89)	49.7	45.2	39.8	86.5	2 (1–12)
ASCEND	Ghia et al. (NCT02970318)	Acalabrutinib	Idelalisib + rituximab/RB	310	68 (32–90)	41.6	15.8	23.5	81.0	2 (1–10)
NR	Burger et al. (NCT02007044)	Ibrutinib + rituximab	Ibrutinib	208	65 (42–83)	38.5	26.9	24.0	59.2	2 (0–5)
DUO	Flinn et al. (NCT02004522)	Duvelisib	Ofatumumab	319	69 (39–90)	35.1	15.4	11.9	44.5	2 (1–10)
NR	Huang et al. (NCT01973387)	Ibrutinib	Rituximab	160	66 (21–87)	72.5	22.5	NR	69.4	2 (1–5)
MURANO	Seymour et al. (NCT02005471)	Venetoclax + rituximab	RB	389	65 (22–85)	12.3	23.7	25.4	73.7	1 (1–5)
NR	Zelenetz et al. (NCT01569295)	Idelalisib + RB	RB	416	63 (56–70)	45.0	18.8	14.2	83.2	2 (1–4)
STUDY119	Jones et al. (NCT01659021)	Idelalisib + ofatumumab	Ofatumumab	261	68 (61–74)	63.6	25.3	14.2	78.6	3 (2–5)
HELIOS	Chanan-Khan et al. (NCT01611090)	Ibrutinib + RB	RB	578	64 (31–86)	37.7	0.0	NR	80.0	2 (1–11)
STUDY116	Furman et al. (NCT01539512)	Idelalisib + Rituximab	Rituximab	220	71 (48–92)	58.6	25.9	NR	76.4	3 (1–12)
RESONATE	Byrd et al. (NCT01578707)	Ibrutinib	Ofatumumab	391	67 (30–88)	56.8	32.5	48.0	68.0	2 (1–13)
ALPINE	Brown et al. (NCT03734016)	Zanubritinib	Ibrutinib	652	67 (35–90)	42.9 (Binet C)	14.6	8.4	77.2	1 (1–12)

*RB* rituximab + bendamustine

### Network meta-analysis of relative effectiveness

Thirteen treatment options were included in the analysis for the overall population: three BTK inhibitors (ibrutinib, acalabrutinib, and zanubrutinib), two BTK inhibitors combined with other treatments (ibrutinib plus rituximab plus bendamustine (RB) and ibrutinib plus rituximab), two PI3K inhibitors (idelalisib and duvelisib), three PI3K inhibitors combined with other treatments (idelalisib plus ofatumumab, idelalisib plus RB, idelalisib plus rituximab), three anti-CD20-based treatment (RB, ofatumumab, and rituximab), and one BCL-2 inhibitors (venetoclax plus rituximab) (Supplementary Fig. S1).

**Fig. 2:**
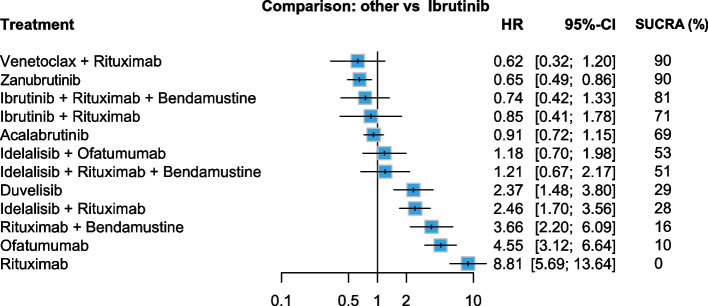
Forest plot of network meta-analysis results for overall population

The relative effectiveness of the treatments for the overall R/R CLL population, derived from our network, is shown in Fig. 2 and Supplementary Table S2. The reference treatment in the forest plot was ibrutinib and the effectiveness of the other therapies was assessed in terms of PFS HRs and their corresponding CIs. Venetoclax plus rituximab and zanubrutinib were the best treatment options. The former had the lowest HR compared with ibrutinib (HR 0.62, 95% CI 0.32–1.20), while the latter had a significantly better HR than ibrutinib (HR 0.65, 95% CI 0.49–0.86). Additionally, a SUCRA value of 90% for both regimens was the highest among the included treatments (Supplementary Fig. S2). The comparator ibrutinib showed a SUCRA value of 61%, which was the 6th rank in the overall population.

**Fig. 3:**
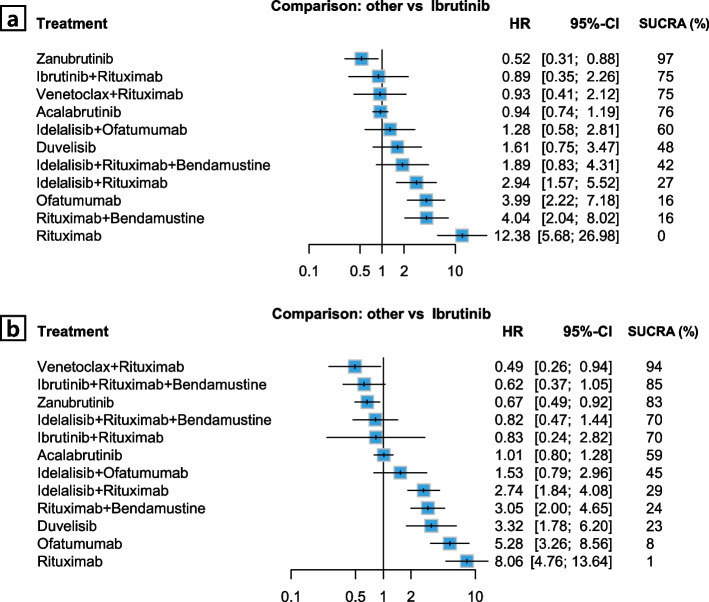
Forest plot of network meta-analysis results by 17p deletion/*TP53* mutation status (**a**) With mutation; (**b**) Without mutation

In the 17p deletion or *TP53* mutation subgroup, ibrutinib plus RB was excluded because the HELIOS trial [10] did not enroll patients with these unfavorable genetic abnormalities. The relative efficacy of the treatments for the 17p deletion/*TP53* mutation subgroup is shown in Fig. 3a and Supplementary Table S3. Zanubrutinib was identified as the best agent, reporting the most favorable and significant HR versus ibrutinib (HR 0.52, 95% CI 0.31–0.88) and the highest SUCRA value of 97% (Supplementary Fig. S3). The second-highest SUCRA was observed for acalabrutinib (76%), followed by ibrutinib plus rituximab (75%) in third place, and ibrutinib (70%) in fifth place. These results indicate the superior efficacy of BTK inhibitors in individuals with unfavorable mutations in this population.

In the subgroup without 17p deletion or *TP53* mutation, venetoclax plus rituximab emerged as the best treatment (Fig. 3b, Supplementary Table S4), with the lowest HR (HR 0.49, 95% CI 0.26–0.94) compared to the reference treatment and the highest SUCRA value (94%) (Supplementary Fig. S4). Zanubrutinib also showed a significantly better PFS than ibrutinib, similar to venetoclax plus rituximab; however, it was ranked as the third-best regimen in this subpopulation.

In addition to the PFS outcomes, we conducted an overall survival analysis. In a forest plot (Supplementary Fig. S5), venetoclax plus rituximab again demonstrated the most favorable overall survival (OS) outcome (HR 0.38, 95% CI 0.16–0.91) relative to ibrutinib, with a SUCRA value of 97%. However, owing to the relatively short follow-up periods in many trials, the 95% confidence intervals for the HRs were broad.

## Discussion

Given the diversity and high molecular heterogeneity of R/R CLL, as well as the absence of validated biomarkers to identify patients who would most benefit from specific treatments, effective systemic therapy remains a crucial aspect of managing this condition. Recently, with the availability of drugs with different mechanisms of action, finding effective treatments for specific conditions has become increasingly important. In this study, we assessed the relative efficacy of various treatments and determined the best interventions for each subgroup using ranking statistics derived from Bayesian simulations. This analysis was conducted for the overall population and for subgroups with and without 17p deletions or *TP53* mutations, as the recommended regimens differ based on the presence of these mutations. A network meta-analysis identified venetoclax plus rituximab and zanubrutinib as the preferred treatment options. Venetoclax plus rituximab performed particularly well in the subgroup without 17p deletion/*TP53* mutation, while zanubrutinib showed better performance in the subgroup with these unfavorable mutations. This analysis provides a comprehensive comparison of the various treatments for R/R CLL and highlights the most effective options for different genetic subgroups.

Several unfavorable prognostic factors have been identified in CLL, including an unmutated *IGHV* gene and cytogenetic abnormalities, such as a complex karyotype, del(17p), and *TP53* mutations. These factors have been shown to be strong predictors of treatment-free interval and overall survival. Specifically, del(17p) and/or *TP53* mutations are associated with shorter treatment-free intervals, poor response to standard chemotherapy, and reduced survival [20].

In our analysis, BTK inhibitors, particularly zanubrutinib, showed remarkable efficacy in the del(17p)/*TP53* subgroup. The RESONATE-17 study highlighted that ibrutinib achieved an objective response rate of 83% and a 24-month PFS rate of 63% in patients with 17p deletions, underscoring its effectiveness in this challenging subgroup [21]. Notably, the ALPINE trial demonstrated that zanubrutinib outperformed ibrutinib, achieving a 24-month PFS rate of 72.6% and 54.6% in patients with del(17p)/*TP53* mutations, respectively [19]. Despite being used in a first-line setting, zanubrutinib also demonstrated high efficacy in treatment-naïve CLL patients with del(17p), with an overall response rate of 94.5% and an estimated 18-month PFS rate of 88.6% [22]. Additionally, the ELEVATE-RR trial showed that acalabrutinib was not inferior to ibrutinib, with robust PFS outcomes in patients with high-risk cytogenetic profiles, including *TP53* mutations [18]. These findings are supported by real-world evidence and pooled analyses [23]. In conclusion, although cautious interpretation is necessary, the use of BTK inhibitors, particularly zanubrutinib, is recommended for subgroups with unfavorable mutation profiles. This finding aligns with the current NCCN guidelines [24], which also highlight BTK inhibitors, including zanubrutinib, as the preferred treatment options for patients with 17p deletion/TP53 mutations. Our results support these guidelines by reinforcing the efficacy of zanubrutinib in these high-risk subgroups, highlighting its potential as a key therapy in personalized treatment strategies for R/R CLL.

Venetoclax combined with rituximab has also shown significant efficacy in the treatment of R/R CLL. The phase 3 MURANO trial provides robust evidence for the benefit of venetoclax plus rituximab, achieving a median PFS of 53.6 months compared to 17.0 months with RB [15]. Notably, an increased PFS was observed across all subgroups, including those with or without del(17p)/*TP53* mutations, indicating the broad applicability of this regimen.

However, based on extended data from the MURANO trial [25], the PFS HR for venetoclax plus rituximab in patients with and without del(17p)/*TP53* mutation was 2.04. The 3-year PFS rate was approximately 51% for those with mutations compared to 80% for those without mutations, highlighting a substantial difference. In contrast, based on the ALPINE data for zanubrutinib, the 3-year PFS rate was 59% in the del(17p)/*TP53* group and 65% in the all-comer population, showing only a slight difference. This suggests that zanubrutinib may be more effective in these high-risk populations, as indicated by our findings.

This meta-analysis had several limitations. First, the line of treatment across the included studies was not entirely consistent, with one study [9] primarily involving elderly and fragile patients. This resulted in some degree of heterogeneity among studies. Due to the unclosed loop structure of the network, it was not possible to perform sensitivity analyses and meta-regression because multiple direct and indirect comparisons were not available. Nonetheless, we ensured homogeneity by separately analyzing patients with significant genetic mutations to provide clinically relevant information. Second, the separate analysis of key genetic mutations may have resulted in our analysis not being sufficiently powered to detect significant differences. Lastly, our analysis focused on the efficacy of PFS and did not include an evaluation of adverse events, which could have provided a more comprehensive understanding of treatment safety profiles.

Despite these limitations, the authors believe that our findings provide important insights for the treatment of R/R CLL. By separately analyzing patients with key genetic mutations, this study highlights the need for personalized treatment approaches based on genetic profiles. These findings provide valuable information for the clinical management of R/R CLL and underscore the importance of tailoring therapeutic strategies.

## Supplementary Information

Supplementary Material 1.

## Data Availability

The data used in this study, both generated and analyzed, were retrieved or estimated from the primary sources cited in this article. The data extracted for the meta-analysis can be obtained upon request from the corresponding author.
