# Supplementary material for: Relative efficacy of systemic treatments for patients with relapsed/refractory chronic lymphocytic leukemia: a network meta-analysis according to 17p deletion/TP53 mutations
Source: Blood Res. 2025 Jan 2;60(1):1. doi: 10.1007/s44313-024-00038-2 (PMC11695543; doi:10.1007/s44313-024-00038-2)

Relative Efficacy of Systemic Treatments for Patients with Relapsed/Refractory Chronic Lymphocytic Leukemia: A Network Meta-Analysis According to 17p Deletion/TP53 Mutations

Jinchul Kim[1], Jinhyun Cho[1], Joo Han Lim[1], and Moon Hee Lee[1]

[1]Department of Hematology-Oncology, Inha University College of Medicine and Hospital, Incheon, Republic of Korea

Methods. Search strategies for PubMed, EMBASE, and Cochrane database

Table S1. Additional baseline characteristics of the included studies

Fig. S1. Network plot of the included treatments

Table S2. Pooled estimates of relative effects of the treatments for overall population

Fig. S2. The surface under the cumulative ranking (SURCA) plot of the treatments for overall population

Table S3. Pooled estimates of relative effects of the treatments for the del(17p)/TP53 mutation subgroup

Fig. S3. The surface under the cumulative ranking (SURCA) plot of the treatments for the del(17p)/TP53 mutation subgroup

Table S4. Pooled estimates of relative effects of the treatments for the non-del(17p)/TP53 mutation subgroup

Fig. S4. The surface under the cumulative ranking (SURCA) plot of the treatments for the non-del(17p)/TP53 mutation subgroup

Fig. S5. Forest plot of network meta-analysis results for overall population in terms of overall survival

Methods: Search strategies for PubMed, EMBASE, and Cochrane database

Pubmed

#1 "chronic lymphocytic leukemia"[All Fields] OR "chronic lymphocytic leukaemia"[All Fields] OR "leukemia, lymphocytic, chronic, b cell"[MeSH Terms] OR (("leukemia"[All Fields] OR "leukaemia"[All Fields]) AND "lymphocytic"[All Fields] AND "chronic"[All Fields]) OR "b-cell chronic lymphocytic leukemia"[All Fields] OR "CLL"[Title/Abstract]

#2 "randomized controlled trial"[Publication Type] OR "randomized controlled trials as topic"[MeSH Terms] OR "randomized controlled trial"[All Fields] OR "randomised controlled trial"[All Fields] OR "RCT"[All Fields] OR (("random allocation"[MeSH Terms] OR "randomized"[All Fields] OR "randomised"[All Fields] OR "randomly"[All Fields]) AND ("clinical trials as topic"[MeSH Terms] OR "trial"[Title/Abstract] OR "study"[Title/Abstract]))

#1 AND #2: 825 results (2023.12.16)

EMBASE

#1 'chronic lymphatic leukemia'/exp/mj OR 'CLL':ab,ti

#2 'randomized controlled trial'/exp

#1 AND #2: 1,087 results (2023.12.16)

Cochrane

#1 chronic lymphocytic leukemia OR CLL

#2 Leukemia, Lymphocytic, Chronic, B-Cell

#3 randomized controlled trial

#4 trials

(#1 OR #2) AND #3 AND #4: 1429 results (2023.12.16)

Table S1. Additional baseline characteristics of the included studies

Trial name	Authors (Trial identifier)	Phase	Year	Journal	Nation	Research	Control	No. of Patients	Male (%)	ECOG PS 2 (%)	Bulky disease (≥ 5cm) (%)	11q del (%)	complex karyotype (%)	Median follow-up duration (m)
ELEVATE-RR	Byrd et al. (NCT02477696)	3	2021	Journal of Clinical Oncology	Multination	Acalabrutinib	Ibrutinib	533	71.1	7.9	49.5	64.2	46.7	40.9
ASCEND	Ghia et al. (NCT02970318)	3	2020	Journal of Clinical Oncology	Multination	Acalabrutinib	Idelalisib+Rituximab /RB	310	67.1	12.9	14.8(>10 cm)	26.8	30.9	16.1
NR	Burger et al. (NCT02007044)	2	2019	Blood	US	Ibrutinib+Rituximab	Ibrutinib	208	70.2	0	NR	20.2	NR	36
DUO	Flinn et al. (NCT02004522)	3	2018	Blood	Multination	Duvelisib	Ofatumumab	319	37.6	5.3	28.5	NA	NR	22.4
NR	Huang et al. (NCT01973387)	3	2018	Cancer Medicine	Multination	Ibrutinib	Rituximab	160	70.6	0	43.8	21.3	NR	17.8
MURANO	Seymour et al. (NCT02005471)	3	2018	New England Journal of Medicine	Multination	Venetoclax+Rituximab	RB	389	73.8	0.8	NR	NR	NR	23.8
NR	Zelenetz et al. (NCT01569295)	3	2017	Lancet Oncology	Multination	Idelalisib+RB	RB	416	76.0	NR	NR	NR	NR	14
STUDY119	Jones et al. (NCT01659021)	3	2017	Lancet Haematology	Multination	Idelalisib+Ofatumumab	Ofatumumab	261	71.3	NR	62.8	NR	NR	16.1
HELIOS	Chanan-Khan et al. (NCT01611090)	3	2015	Lancet Oncology	Multination	Ibrutinib+RB	RB	578	66.1	0	56.1	26.3	NR	17
STUDY116	Furman et al. (NCT01539512)	3	2014	New England Journal of Medicine	Multination	Idelalisib+Rituximab	Rituximab	220	65.5	NR	NR	NR	NR	NR
RESONATE	Byrd et al. (NCT01578707)	3	2014	New England Journal of Medicine	Multination	Ibrutinib	Ofatumumab	391	68.0	0	57.5	31.2	NR	9.4
ALPINE	Brown et al. (NCT03734016)	3	2023	New England Journal of Medicine	Multination	Zanubritinib	Ibrutinib	652	68.3	NR	38.2	27.5	19.3	29.6

RB, Rituximab+Bendamustine

Fig. S1. Network plot of the included treatments

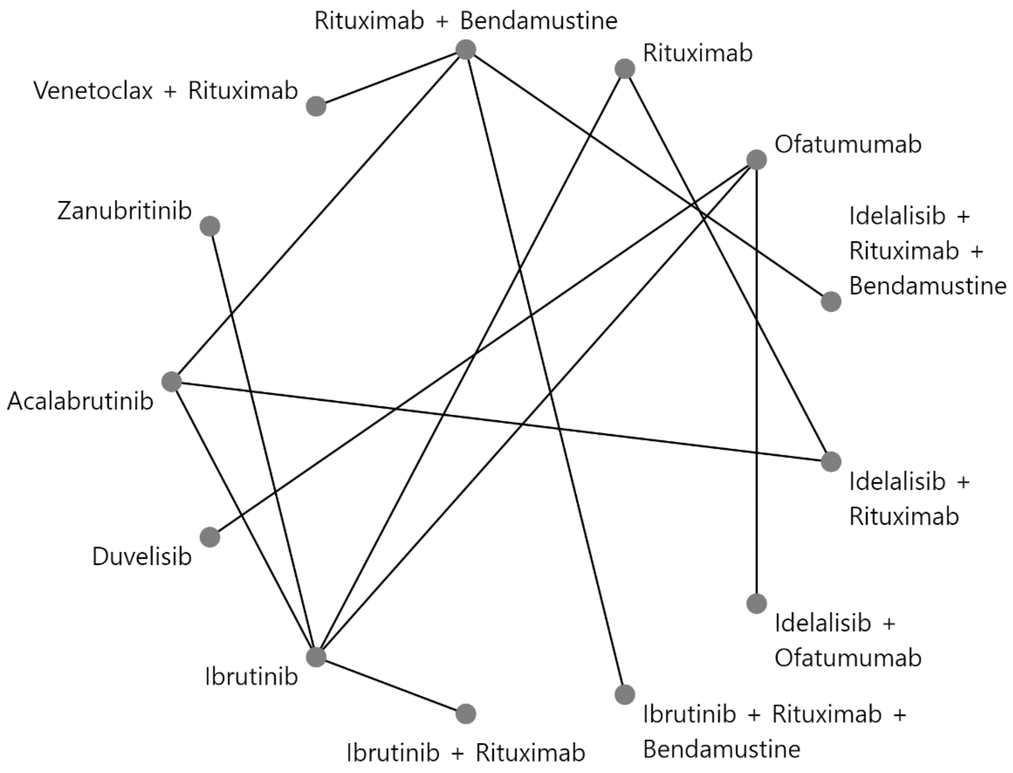

Table S2. Pooled estimates of relative effects of the treatments for overall population

	acalabrutinib	duvelisib	ibrutinib	ibrutinib+rituximab	ibrutinib+RB	idelalisib+ofatumumab	idelalisib+rituximab	idelalisib+RB	ofatumumab	rituximab	RB	venetoclax+rituximab	zanubritinib
acalabrutinib	acalabrutinib	2.58 (1.49, 4.41)	1.09 (0.86, 1.38)	0.94 (0.42, 2.07)	0.82 (0.48, 1.4)	1.29 (0.73, 2.32)	2.7 (1.97, 3.72)	1.31 (0.76, 2.31)	4.96 (3.18, 7.75)	9.59 (6.06, 15.26)	4.02 (2.55, 6.29)	0.68 (0.37, 1.23)	0.71 (0.49, 1.02)
duvelisib	0.39 (0.23, 0.67)	duvelisib	0.42 (0.26, 0.69)	0.36 (0.15, 0.89)	0.32 (0.15, 0.67)	0.5 (0.32, 0.82)	1.05 (0.57, 1.88)	0.51 (0.24, 1.1)	1.93 (1.43, 2.56)	3.72 (1.93, 7.21)	1.56 (0.77, 3.15)	0.26 (0.12, 0.59)	0.27 (0.16, 0.48)
ibrutinib	0.92 (0.72, 1.16)	2.36 (1.45, 3.8)	ibrutinib	0.86 (0.41, 1.85)	0.75 (0.42, 1.36)	1.19 (0.7, 2.05)	2.47 (1.67, 3.57)	1.2 (0.67, 2.19)	4.54 (3.08, 6.62)	8.79 (5.65, 13.66)	3.66 (2.22, 6.2)	0.62 (0.32, 1.20)	0.65 (0.49, 0.86)
ibrutinib+rituximab	1.06 (0.48, 2.36)	2.76 (1.12, 6.81)	1.16 (0.54, 2.44)	ibrutinib+rituximab	0.87 (0.34, 2.29)	1.39 (0.53, 3.49)	2.89 (1.22, 6.69)	1.4 (0.55, 3.61)	5.33 (2.27, 12.51)	10.13 (4.22, 24.33)	4.27 (1.72, 10.55)	0.72 (0.27, 1.92)	0.75 (0.34, 1.68)
ibrutinib+RB	1.22 (0.71, 2.1)	3.15 (1.48, 6.74)	1.34 (0.74, 2.4)	1.15 (0.44, 2.91)	ibrutinib+RB	1.59 (0.73, 3.48)	3.29 (1.77, 6.32)	1.61 (1.06, 2.47)	6.06 (3.06, 12.27)	11.69 (5.77, 23.95)	4.9 (3.64, 6.67)	0.83 (0.5, 1.37)	0.87 (0.45, 1.65)
idelalisib+ofatumumab	0.77 (0.43, 1.38)	1.99 (1.22, 3.14)	0.84 (0.49, 1.44)	0.72 (0.29, 1.87)	0.63 (0.29, 1.37)	idelalisib+ofatumumab	2.1 (1.1, 4.04)	1.01 (0.46, 2.26)	3.84 (2.62, 5.49)	7.46 (3.76, 14.55)	3.1 (1.52, 6.39)	0.52 (0.22, 1.18)	0.55 (0.3, 1)
idelalisib+rituximab	0.37 (0.27, 0.51)	0.95 (0.53, 1.75)	0.4 (0.28, 0.6)	0.35 (0.15, 0.82)	0.3 (0.16, 0.56)	0.48 (0.25, 0.91)	idelalisib+rituximab	0.49 (0.26, 0.93)	1.83 (1.11, 3.12)	3.56 (2.23, 5.61)	1.49 (0.85, 2.59)	0.25 (0.13, 0.5)	0.26 (0.17, 0.42)
idelalisib+RB	0.76 (0.43, 1.31)	1.96 (0.91, 4.09)	0.83 (0.46, 1.5)	0.71 (0.28, 1.8)	0.62 (0.41, 0.94)	0.99 (0.44, 2.17)	2.05 (1.08, 3.89)	idelalisib+RB	3.79 (1.87, 7.51)	7.27 (3.52, 15.35)	3.04 (2.31, 4.05)	0.52 (0.31, 0.84)	0.54 (0.28, 1.05)
ofatumumab	0.2 (0.13, 0.31)	0.52 (0.39, 0.7)	0.22 (0.15, 0.32)	0.19 (0.08, 0.44)	0.17 (0.08, 0.33)	0.26 (0.18, 0.38)	0.55 (0.32, 0.9)	0.26 (0.13, 0.54)	ofatumumab	1.94 (1.1, 3.39)	0.81 (0.44, 1.52)	0.14 (0.06, 0.29)	0.14 (0.09, 0.23)
rituximab	0.1 (0.07, 0.16)	0.27 (0.14, 0.52)	0.11 (0.07, 0.18)	0.1 (0.04, 0.24)	0.09 (0.04, 0.17)	0.13 (0.07, 0.27)	0.28 (0.18, 0.45)	0.14 (0.07, 0.28)	0.52 (0.29, 0.91)	rituximab	0.42 (0.22, 0.8)	0.07 (0.03, 0.15)	0.07 (0.04, 0.12)
RB	0.25 (0.16, 0.39)	0.64 (0.32, 1.29)	0.27 (0.16, 0.45)	0.23 (0.09, 0.58)	0.2 (0.15, 0.27)	0.32 (0.16, 0.66)	0.67 (0.39, 1.17)	0.33 (0.25, 0.43)	1.24 (0.66, 2.29)	2.39 (1.25, 4.58)	RB	0.17 (0.11, 0.25)	0.18 (0.1, 0.32)
venetoclax+rituximab	1.47 (0.81, 2.73)	3.8 (1.69, 8.63)	1.61 (0.84, 3.1)	1.38 (0.52, 3.74)	1.21 (0.73, 2.01)	1.92 (0.85, 4.47)	3.99 (2.01, 7.84)	1.94 (1.19, 3.19)	7.33 (3.46, 15.52)	14.05 (6.69, 30.6)	5.91 (3.95, 8.79)	venetoclax+rituximab	1.05 (0.53, 2.07)
zanubritinib	1.41 (0.98, 2.04)	3.64 (2.1, 6.27)	1.54 (1.16, 2.04)	1.33 (0.6, 2.91)	1.15 (0.6, 2.22)	1.83 (1, 3.37)	3.83 (2.39, 6.03)	1.86 (0.95, 3.55)	6.99 (4.38, 11.29)	13.58 (8.08, 22.81)	5.66 (3.17, 10.05)	0.95 (0.48, 1.9)	zanubritinib

RB, Rituximab+Bendamustine

Comparison of the included interventions: progression-free survival presented with hazard ratio (95% credible interval). Each cell gives the effect of the column-defining intervention relative to the row-defining intervention.

Fig. S2. The surface under the cumulative ranking (SURCA) plot of the treatments for overall population

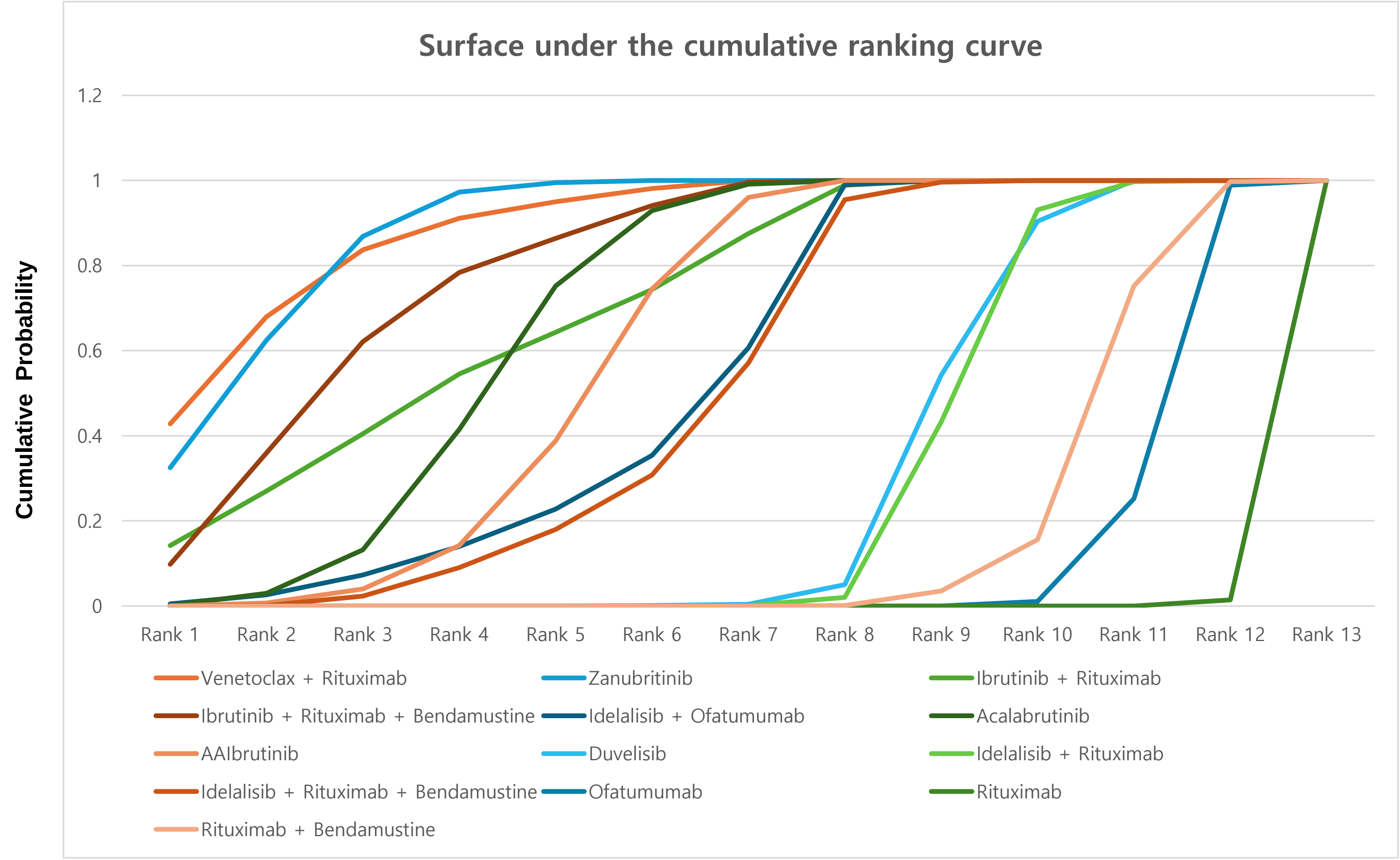

Table S3. Pooled estimates of relative effects of the treatments for the del(17p)/TP53 mutation subgroup

	acalabrutinib	duvelisib	ibrutinib	ibrutinib+rituximab	idelalisib+ofatumumab	idelalisib+rituximab	idelalisib+RB	ofatumumab	rituximab	RB	venetoclax+rituximab	zanubrutinib
acalabrutinib	acalabrutinib	1.71 (0.75, 3.76)	1.07 (0.85, 1.35)	0.94 (0.37, 2.4)	1.38 (0.58, 3.13)	3.14 (1.71, 5.56)	2.05 (0.93, 4.56)	4.3 (2.24, 7.99)	13.44 (6.17, 28.83)	4.34 (2.28, 8.53)	1 (0.45, 2.26)	0.55 (0.31, 0.99)
duvelisib	0.58 (0.27, 1.33)	duvelisib	0.62 (0.29, 1.38)	0.55 (0.17, 1.85)	0.8 (0.38, 1.73)	1.85 (0.7, 4.9)	1.2 (0.4, 3.64)	2.52 (1.49, 4.21)	7.79 (2.6, 23.31)	2.55 (0.92, 7.23)	0.59 (0.18, 1.82)	0.32 (0.12, 0.85)
ibrutinib	0.94 (0.74, 1.18)	1.61 (0.72, 3.4)	ibrutinib	0.88 (0.34, 2.19)	1.29 (0.56, 2.88)	2.95 (1.58, 5.35)	1.92 (0.84, 4.3)	4.02 (2.21, 7.11)	12.47 (5.75, 27.6)	4.07 (2.02, 8.34)	0.94 (0.41, 2.18)	0.52 (0.31, 0.88)
ibrutinib+rituximab	1.06 (0.42, 2.72)	1.81 (0.54, 5.97)	1.13 (0.46, 2.9)	ibrutinib+rituximab	1.46 (0.42, 5.07)	3.3 (1.09, 10.2)	2.14 (0.66, 7.29)	4.52 (1.56, 13.61)	13.91 (4.43, 50.62)	4.57 (1.49, 14.36)	1.07 (0.32, 3.65)	0.58 (0.21, 1.64)
idelalisib+ofatumumab	0.73 (0.32, 1.73)	1.25 (0.58, 2.66)	0.77 (0.35, 1.79)	0.68 (0.2, 2.41)	idelalisib+ofatumumab	2.29 (0.81, 6.4)	1.51 (0.48, 4.78)	3.1 (1.77, 5.48)	9.72 (3.21, 30.11)	3.16 (1.1, 9.43)	0.73 (0.23, 2.37)	0.4 (0.15, 1.09)
idelalisib+rituximab	0.32 (0.18, 0.58)	0.54 (0.2, 1.43)	0.34 (0.19, 0.63)	0.3 (0.1, 0.92)	0.44 (0.16, 1.24)	idelalisib+rituximab	0.65 (0.24, 1.77)	1.36 (0.6, 3.27)	4.3 (2.03, 8.95)	1.38 (0.59, 3.43)	0.32 (0.12, 0.9)	0.18 (0.08, 0.4)
idelalisib+RB	0.49 (0.22, 1.07)	0.83 (0.27, 2.52)	0.52 (0.23, 1.19)	0.47 (0.14, 1.53)	0.66 (0.21, 2.09)	1.53 (0.57, 4.15)	idelalisib+RB	2.1 (0.75, 5.58)	6.57 (2.21, 19.33)	2.12 (1.4, 3.29)	0.49 (0.26, 0.92)	0.27 (0.1, 0.72)
ofatumumab	0.23 (0.13, 0.45)	0.4 (0.24, 0.67)	0.25 (0.14, 0.45)	0.22 (0.07, 0.64)	0.32 (0.18, 0.57)	0.73 (0.31, 1.67)	0.48 (0.18, 1.33)	ofatumumab	3.13 (1.18, 8.39)	1.02 (0.42, 2.52)	0.23 (0.08, 0.67)	0.13 (0.06, 0.29)
rituximab	0.07 (0.03, 0.16)	0.13 (0.04, 0.38)	0.08 (0.04, 0.17)	0.07 (0.02, 0.23)	0.1 (0.03, 0.31)	0.23 (0.11, 0.49)	0.15 (0.05, 0.45)	0.32 (0.12, 0.85)	rituximab	0.32 (0.12, 0.91)	0.07 (0.03, 0.24)	0.04 (0.02, 0.11)
RB	0.23 (0.12, 0.44)	0.39 (0.14, 1.09)	0.25 (0.12, 0.49)	0.22 (0.07, 0.67)	0.32 (0.11, 0.91)	0.72 (0.29, 1.7)	0.47 (0.3, 0.71)	0.98 (0.4, 2.4)	3.1 (1.1, 8.23)	RB	0.23 (0.14, 0.37)	0.13 (0.05, 0.31)
venetoclax+rituximab	1 (0.44, 2.25)	1.69 (0.55, 5.46)	1.07 (0.46, 2.46)	0.94 (0.27, 3.13)	1.37 (0.42, 4.42)	3.15 (1.11, 8.57)	2.04 (1.08, 3.88)	4.28 (1.5, 12.07)	13.43 (4.22, 39.69)	4.35 (2.7, 7.02)	venetoclax+rituximab	0.55 (0.2, 1.53)
zanubrutinib	1.82 (1.01, 3.23)	3.11 (1.17, 8.09)	1.95 (1.13, 3.35)	1.71 (0.61, 4.8)	2.53 (0.92, 6.86)	5.71 (2.52, 12.84)	3.74 (1.39, 10)	7.8 (3.46, 17.83)	24.3 (9.52, 63.12)	7.98 (3.25, 19.05)	1.83 (0.65, 4.88)	zanubrutinib

RB, Rituximab+Bendamustine

Comparison of the included interventions: progression-free survival presented with hazard ratio (95% credible interval). Each cell gives the effect of the column-defining intervention relative to the row-defining intervention.

Fig. S3. The surface under the cumulative ranking (SURCA) plot of the treatments for the del(17p)/TP53 mutation subgroup

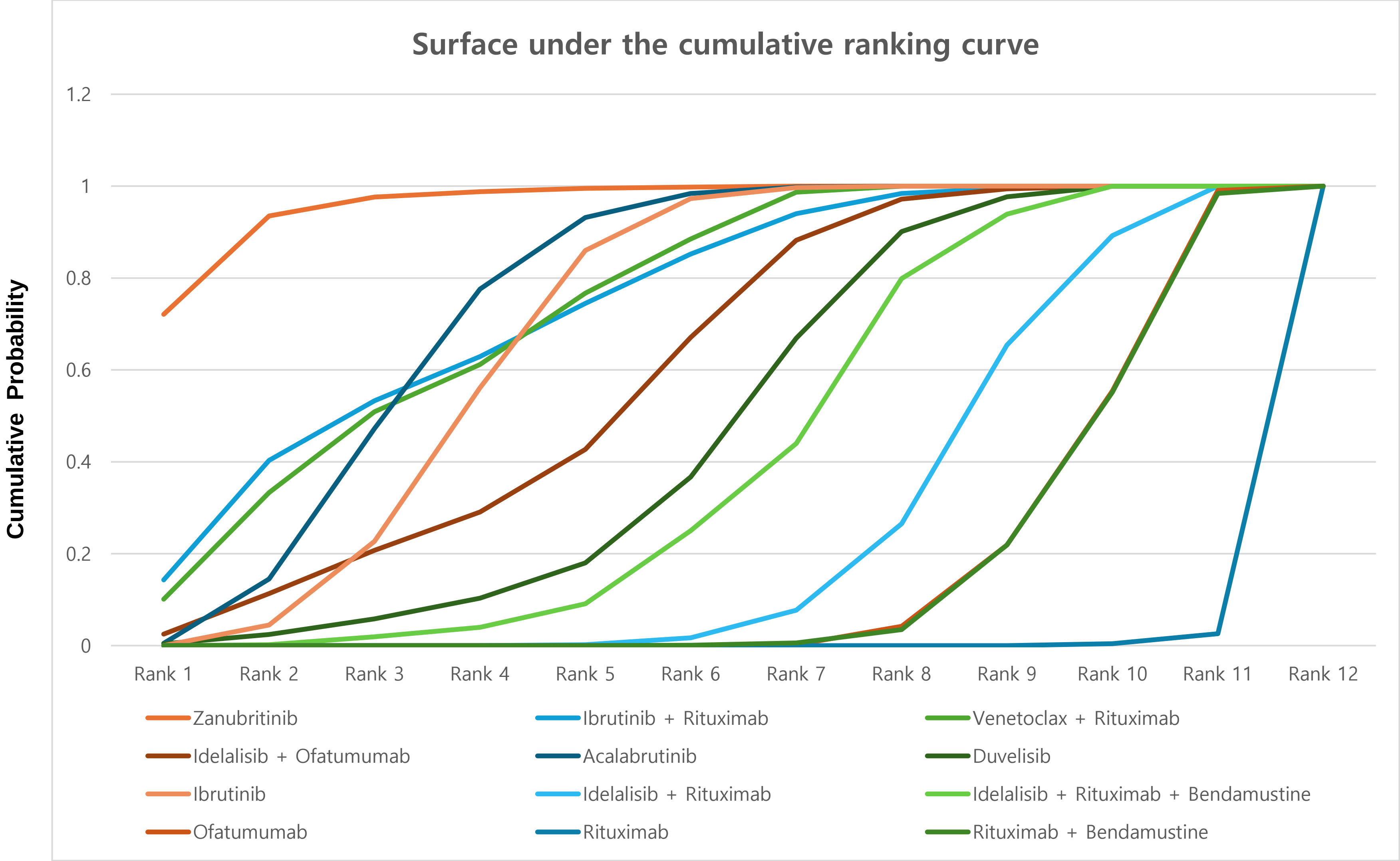

Table S4. Pooled estimates of relative effects of the treatments for the non-del(17p)/TP53 mutation subgroup

	acalabrutinib	duvelisib	ibrutinib	ibrutinib+rituximab	ibrutinib+RB	idelalisib+ofatumumab	idelalisib+rituximab	idelalisib+RB	ofatumumab	rituximab	RB	venetoclax+rituximab	zanubritinib
acalabrutinib	acalabrutinib	3.31 (1.69, 6.52)	0.99 (0.78, 1.25)	0.81 (0.24, 2.91)	0.61 (0.38, 0.98)	1.52 (0.75, 3.13)	2.73 (1.96, 3.84)	0.82 (0.47, 1.39)	5.24 (3.08, 9.03)	8.11 (4.6, 14.15)	3.01 (2.1, 4.28)	0.48 (0.26, 0.88)	0.67 (0.44, 1)
duvelisib	0.3 (0.15, 0.59)	duvelisib	0.3 (0.16, 0.56)	0.25 (0.06, 0.99)	0.18 (0.08, 0.42)	0.46 (0.25, 0.87)	0.83 (0.39, 1.73)	0.25 (0.1, 0.57)	1.58 (1.06, 2.36)	2.44 (1.07, 5.69)	0.91 (0.42, 1.97)	0.15 (0.06, 0.37)	0.2 (0.1, 0.41)
ibrutinib	1.01 (0.8, 1.29)	3.32 (1.78, 6.3)	ibrutinib	0.82 (0.24, 2.84)	0.62 (0.36, 1.05)	1.54 (0.78, 3.04)	2.76 (1.82, 4.14)	0.82 (0.46, 1.45)	5.27 (3.22, 8.56)	8.16 (4.75, 13.81)	3.05 (1.95, 4.64)	0.49 (0.26, 0.94)	0.67 (0.49, 0.93)
ibrutinib+rituximab	1.23 (0.34, 4.25)	4.03 (1.01, 16.01)	1.23 (0.35, 4.16)	ibrutinib+rituximab	0.75 (0.19, 2.75)	1.86 (0.46, 7.55)	3.36 (0.92, 12.27)	1 (0.24, 3.74)	6.48 (1.75, 24.09)	9.93 (2.58, 37.86)	3.72 (0.97, 13.03)	0.6 (0.14, 2.29)	0.82 (0.23, 2.9)
ibrutinib+RB	1.63 (1.02, 2.62)	5.42 (2.38, 12.23)	1.62 (0.96, 2.81)	1.33 (0.36, 5.3)	ibrutinib+RB	2.48 (1.07, 6.09)	4.44 (2.52, 7.95)	1.33 (0.82, 2.2)	8.59 (4.31, 17.58)	13.1 (6.39, 28.02)	4.92 (3.59, 6.65)	0.79 (0.44, 1.4)	1.1 (0.58, 2.05)
idelalisib+ofatumumab	0.66 (0.32, 1.34)	2.16 (1.14, 3.98)	0.65 (0.33, 1.28)	0.54 (0.13, 2.18)	0.4 (0.16, 0.94)	idelalisib+ofatumumab	1.8 (0.8, 3.94)	0.54 (0.22, 1.27)	3.44 (2.15, 5.52)	5.31 (2.31, 12.55)	1.99 (0.87, 4.37)	0.31 (0.12, 0.83)	0.44 (0.2, 0.91)
idelalisib+rituximab	0.37 (0.26, 0.51)	1.21 (0.58, 2.54)	0.36 (0.24, 0.55)	0.3 (0.08, 1.08)	0.23 (0.13, 0.4)	0.56 (0.25, 1.24)	idelalisib+rituximab	0.3 (0.16, 0.56)	1.91 (1.03, 3.62)	2.97 (1.65, 5.31)	1.1 (0.67, 1.79)	0.18 (0.09, 0.35)	0.24 (0.14, 0.41)
idelalisib+RB	1.22 (0.72, 2.11)	4.04 (1.75, 9.97)	1.22 (0.69, 2.19)	1 (0.27, 4.12)	0.75 (0.46, 1.22)	1.86 (0.79, 4.63)	3.37 (1.78, 6.25)	idelalisib+RB	6.42 (3.06, 13.97)	9.88 (4.6, 22.1)	3.69 (2.53, 5.49)	0.59 (0.31, 1.1)	0.82 (0.42, 1.6)
ofatumumab	0.19 (0.11, 0.32)	0.63 (0.42, 0.94)	0.19 (0.12, 0.31)	0.15 (0.04, 0.57)	0.12 (0.06, 0.23)	0.29 (0.18, 0.47)	0.52 (0.28, 0.97)	0.16 (0.07, 0.33)	ofatumumab	1.55 (0.74, 3.16)	0.57 (0.3, 1.09)	0.09 (0.04, 0.21)	0.13 (0.07, 0.23)
rituximab	0.12 (0.07, 0.22)	0.41 (0.18, 0.94)	0.12 (0.07, 0.21)	0.1 (0.03, 0.39)	0.08 (0.04, 0.16)	0.19 (0.08, 0.43)	0.34 (0.19, 0.61)	0.1 (0.05, 0.22)	0.64 (0.32, 1.35)	rituximab	0.37 (0.19, 0.72)	0.06 (0.02, 0.13)	0.08 (0.04, 0.15)
RB	0.33 (0.23, 0.48)	1.09 (0.51, 2.38)	0.33 (0.22, 0.51)	0.27 (0.08, 1.03)	0.2 (0.15, 0.28)	0.5 (0.23, 1.15)	0.91 (0.56, 1.5)	0.27 (0.18, 0.39)	1.75 (0.92, 3.32)	2.69 (1.39, 5.33)	RB	0.16 (0.1, 0.26)	0.22 (0.13, 0.38)
venetoclax+rituximab	2.07 (1.13, 3.83)	6.88 (2.73, 17.27)	2.07 (1.08, 4.06)	1.67 (0.44, 7.22)	1.27 (0.71, 2.28)	3.18 (1.21, 8.07)	5.66 (2.86, 11.55)	1.69 (0.91, 3.25)	10.93 (4.87, 24.91)	16.83 (7.41, 40.18)	6.27 (3.81, 10.44)	venetoclax+rituximab	1.39 (0.67, 2.88)
zanubritinib	1.5 (1, 2.26)	4.97 (2.45, 10.04)	1.49 (1.07, 2.06)	1.21 (0.34, 4.41)	0.91 (0.49, 1.72)	2.27 (1.1, 4.91)	4.09 (2.46, 6.98)	1.22 (0.63, 2.39)	7.86 (4.41, 13.99)	12.24 (6.46, 22.98)	4.52 (2.63, 7.83)	0.72 (0.35, 1.48)	zanubritinib

RB, Rituximab+Bendamustine

Comparison of the included interventions: progression-free survival presented with hazard ratio (95% credible interval). Each cell gives the effect of the column-defining intervention relative to the row-defining intervention.

Fig. S4. The surface under the cumulative ranking (SURCA) plot of the treatments for the non-del(17p)/TP53 mutation subgroup

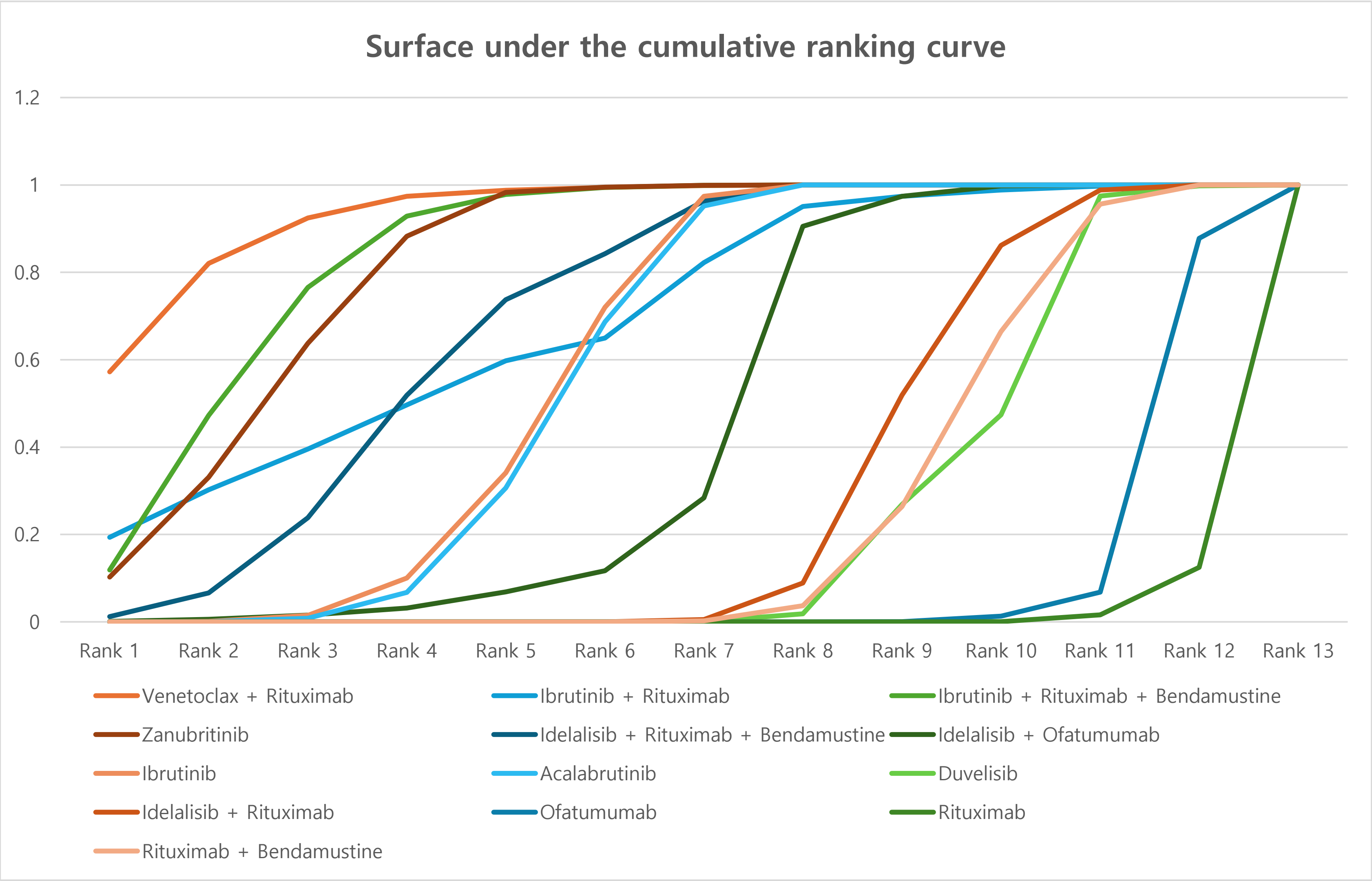

Fig. S5. Forest plot of network meta-analysis results for overall population in terms of overall survival

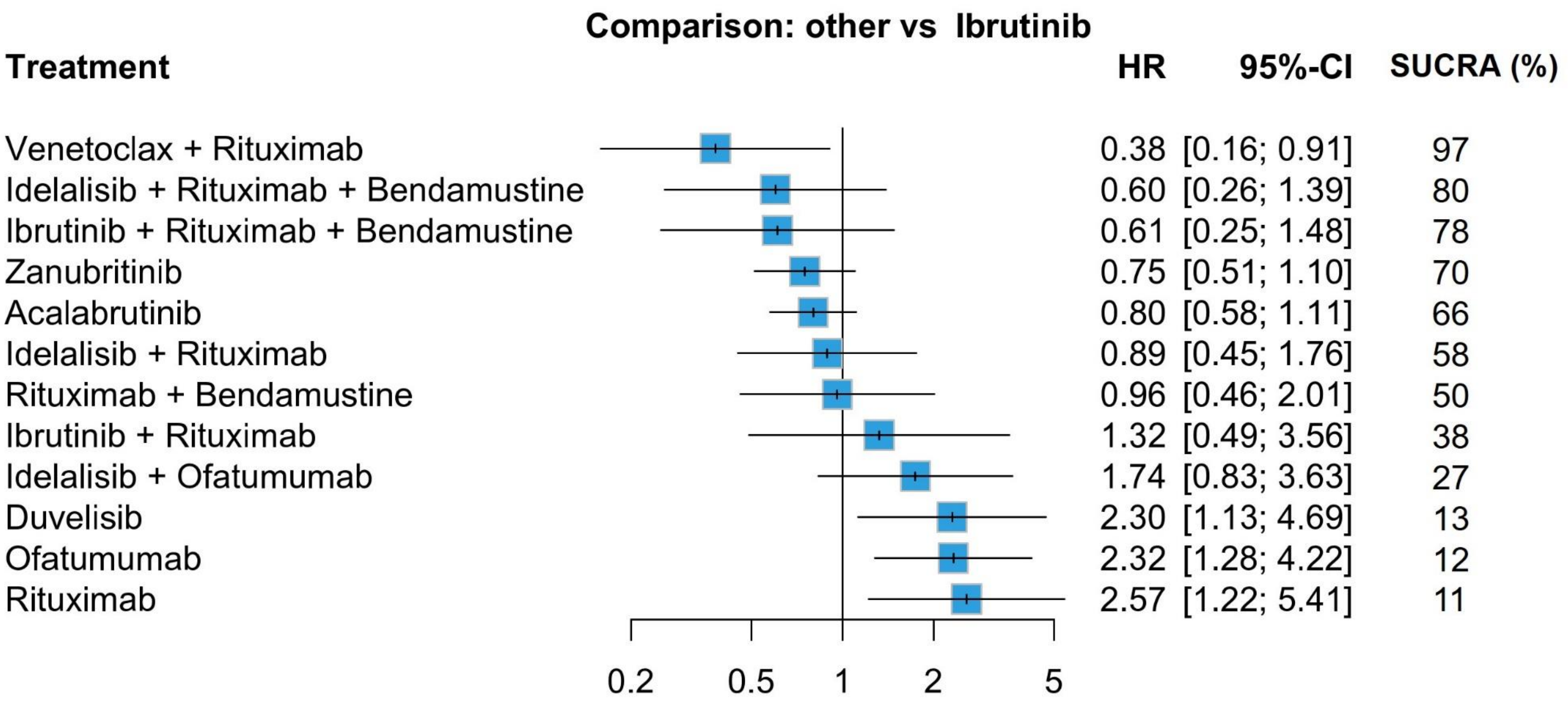

Supplement: Supplementary file 1 — Supplementary Material 1. [file 44313_2024_38_MOESM1_ESM.pdf]
